# Effect of Different Host Plants on Nutritional Indices of the Pod Borer, *Helicoverpa armigera*

**DOI:** 10.1673/031.012.5501

**Published:** 2012-04-18

**Authors:** S.A. Hemati, B. Naseri, G. Nouri Ganbalani, H. Rafiee Dastjerdi, A. Golizadeh

**Affiliations:** Department of Plant Protection, Faculty of Agriculture, University of Mohaghegh Ardabili, Ardabil, Iran

**Keywords:** antibiosis, feeding performance, gram pod borer, Noctuidae

## Abstract

Nutritional indices of *Helicoverpa armigera* (Hübner) (Lepidoptera: Noctuidae) on different host plants including chickpea (cultivars Arman, Hashem, Azad, and Binivich), common bean (cultivar Khomein), white kidney bean (cultivar Dehghan), red kidney bean (cultivar Goli), cowpea (cultivar Mashhad), tomato (cultivar Meshkin) and potato (cultivars Agria and Satina) were studied under laboratory conditions (25 ± 1 °C, 65 ± 5% RH, 16:8 L:D). Third instar larvae reared on potato Agria showed the highest efficiency of conversion of digested food (ECD) and efficiency of conversion of ingested food (ECI) (50.800 é 0.104% and 13.630 ± 0.016%, respectively). Approximate digestibility (AD) values of the fourth instar larvae were highest (92.651 ± 0.004%) and lowest (57.140 — 0.049%) on chickpea Azad and potato Agria, respectively. The fifth instar larvae fed on tomato Meshkin and white kidney bean Dehghan had the highest consumption index (CI) (3.717 ± 0.091) and relative consumption rate (RCR) (1.620 ± 0.074), respectively. Whole larval instars showed the highest ECI and ECD values on potatoes Satina (14.640 ± 0.014%) and Agria (21.380 ± 0.015%), respectively, and the lowest of both values on tomato Meshkin (ECI: 5.748 ± 0.002% and ECD: 7.341 ± 0.002%). The results of nutritional indices and the cluster analysis indicated that tomato Meshkin was an unsuitable host for feeding of *H. armigera.*

## Introduction

The gram or noctuid pod borer, *Helicoverpa armigera* (Hübner) (Lepidoptera: Noctuidae), is a voracious feeder pest that infests over 100 plant species including widespread and economically important crops such as cotton, maize, tobacco, pigeonpea, chickpea, and tomato (Talekar et al. 2006). The preference of *H. armigera* to feed on the harvestable parts of host plants, along with its high polyphagy and mobility, broad geographical variety, migratory potential, facultative diapause, high fecundity, and tendency to develop resistance to insecticides lead to its status as an important crop pest (Fitt 1989; Zalucki 1991; Anonymous 2000). Increases in intensive crop production technologies and concomitant insecticide resistance due to use of broad spectrum insecticides, as well as continuous accessibility of preferred food plants have favored *H. armigera* to become a major pest of crops (Fitt et al. 1995; Naseri et al. 2009; Fathipour and Naseri 2011). The present research was carried out in order to identify alternative methods of chemical control for *H. armigera.*

Different nutritive values of host plants may influence the rate of development of *H. armigera* larvae, thus affecting the population dynamics of this pest (Ruan and Wu 2001). The contributions of host plants to developing generations of *H. armigera* are clear and well—understood. The availability of different host plants plays an essential role in causing population outbreaks for polyphagous insects (Singh and Parihar 1988). The quality and quantity of food consumed may increasingly affect growth, development, and reproduction of insects (Scriber and Slansky 1981).

Host plant resistance among crop plants is a major part of integrated pest management (IPM). It is relatively constant, cheap, non— polluting, and is compatible with other methods of pest control. Developing resistant cultivars to *H. armigera* would supply an effective complementary approach in IPM to reduce the extent of losses caused by this pest (Sachan 1990; Jallow et al. 2004).

Since various host plant cultivars tested in our research (including bean, chickpea, tomato, and potato cultivars) have different nutritional values for *H. armigera* larvae, we hypothesize that the larvae will accumulate body mass more efficiently on some host plant cultivars than the others. For example, in light of the higher protein content in bean and chickpea cultivars, we hypothesize that *H. armigera* larvae will have higher efficiency of conversion of ingested and digested food on bean and chickpea cultivars than on tomato and potato cultivars.

Despite the economic importance of *H. armigera*, little published information exists on the nutritional indices of this pest on different host plants; however, some related studies have been done on the influence of host plants apart from those tested in our research on feeding indices of *H. armigera.* The efficiency of food utilization by *Helicoverpa zea* raised on artificial diets or green beans (Cohen and Patana 1984) indicated that the efficiency of food utilization for bean—fed larvae was higher than diet—fed larvae both in terms of dry matter conversion and energy conversion. A study on the growth and food consumption of *H. zea* larvae on foliage of wild and cultivated tomatoes (Farrar and Kennedy 1987) showed that both resistant and susceptible foliage was found to contain factors that increased larval mortality, reduced larval weigh, reduced consumption rate, and reduced growth efficiency. Research on morpho—physical factors affecting consumption and coefficient of utilization of *H. armigera* (Ashfaq et al. 2003) demonstrated that preference on the basis of consumption and coefficient of utilization was highest on sorghum than on the other hosts. A study of nutritional indices of *H. armigera* on different soybean varieties by Naseri et al. (2010) showed that varieties M4, Sahar, and JK were partially resistant to *H. armigera.* Working on feeding indices of *H. armigera* reared on seeds of five different maize hybrids, Arghand et al. (2011) reported that hybrid SC700 was partially resistant to this pest. The previous works conducted by the above—mentioned authors, however, did not consider all of the nutritional indices of *H. armigera*, and did not compare the important indices of larvae within host plants. Furthermore, the host plants examined in the current study were different from those that have been previously tested. The goal of this research was to compare food utilization indices for *H. armigera* on different host plants to determine how these indices changed after an additional instar, and to understand response of this pest to different host plants with varying nutritional values. The outcome of this research, along with the findings of previous studies, could allow for the creation of a comprehensive plan for an integrated pest management program for *H. armigera* on different host plants.

## Materials and Methods

### Plant sources

Seeds of different host plants including chickpea (*Cicer arietinum* L.) (cultivars Arman, Hashem, Azad, and Binivich), common bean (*Phaseolus vulgaris* L.) (cultivar Khomein), white kidney bean (*P.* *vulgaris*) (cultivar Dehghan), red kidney bean (*P. vulgaris)* (cultivar Goli), cowpea (*Vigna sinensis* L.) (cultivar Mashhad), tomato (*Lycopersicon esculentum* Mill) (cultivar Meshkin), and potato (*Solanum tuberosum* L.) (cultivars Agria and Satina) were provided from the Plant and Seed Modification Research Institute in Karaj, Iran. They were planted in the research field of the University of Mohaghegh Ardabili located in Ardabil, Iran, in May 2010. The experiments were started in early July 2010 after the host plants reached the reproductive stage (the green same—size of terminal pods for beans, chickpea, and cowpea; immature green fruit for tomato) and had younger same—sized leaves, but for potatoes the leaves were not fully expanded . For this research the leaves, pods, and fruits of the various host plants were transferred to a growth chamber at 25 ± 1 °C, 65 ± 5% RH, and 16:8 L:D. Examinations were conducted during the morning and afternoon mid—July to mid—September 2010. According to our observations on feeding behavior of the larvae *H. armigera*, the leaves of different hosts were used for feeding of first and second larval instars, and the green pods (chickpea, common bean, white kidney bean, red kidney bean, and cowpea), fruit (tomato), and leaf (potato) were used for feeding of the third to fifth larval instars, as reported by Green et al. (2002) and Naseri et al. (2009, 2010).

### Laboratory colony

*Helicoverpa armigera* larvae used in the experiment were obtained from a laboratory colony maintained on a defined cowpea—based artificial diet at the Tabriz University Department of Plant Protection. The artificial diet contained: powdered cowpea seed (250 g), wheat germ (30 g), yeast (35 g), sorbic acid (1.1 g), ascorbic acid (3.5 g), sunflower oil (5 ml), agar (14 g), methyl-p-hydroxy benzoate (2.2 g), formaldehyde 37% (2.5 mL), and distilled water (650 mL) (Shorey and Hale 1965). Stock culture was initiated on different host plants in a growth chamber (25 — 1 °C, 65 — 5% RH, 16:8 L:D).

### Experiments

Neonate larvae were gathered from the stock culture and separated into five replicates (10 larvae in each) and transferred into plastic containers (diameter 19.5 cm, depth 7.5 cm) with a hole covered by a mesh net for aeration, containing the fresh leaves of each examined plant. The petioles of detached leaves were wrapped in water—soaked cotton to maintain freshness. Nutritional indices were determined using third to fifth instars, as they were easier to measure than the primary instars. The first and second instar larvae were reared in groups until the third instar, after which they were divided onto individual plastic plates (diameter 8 cm, depth 1 cm) to prevent cannibalistic behavior (Twine 1971). For pre—pupation and pupation, fifth instar larvae were kept in small plastic tubes (diameter 2 cm, depth 5 cm).

To determine weight gain, food utilization, and feces produced by the larvae, a gravimetric method was used. Nutritional indices were evaluated on the basis of dry weight. After measuring the weight of third instar larvae, specimens were set up on the leaves (potato), pods (chickpea, common bean, white kidney bean, red kidney bean, and cowpea), and fruits (tomato), and larval weight was recorded daily for two weeks before and after feeding until they stopped feeding and reached the pre—pupal stage. The initial fresh food and the food and feces remaining at the end of each experiment were weighed daily. The quantity of food ingested was determined by subtracting the diet remaining at the end of each experiment from the total weight of fresh diet supplied. The weight of feces produced by the larvae fed on different host plants was recorded every day. To obtain the percentage of dry weight of the food, feces, and larvae, 20 specimens for each were weighed, oven—dried (48 hours at 60 °C), and subsequently re—weighed. In this study, “natural losses” including biomass changes in substances other than water (carbon dioxide, volatile materials, microbial decay products, etc.) were not measured.

Nutritional indices were calculated using formulae described by Waldbauer (1968): consumption index (CI) = E/A; approximate digestibility (AD) = E—F/E; efficiency of conversion of ingested food (ECI) = P/E; efficiency of conversion of digested food (ECD) = P/E—F; relative consumption rate (RCR) = E/A*T; and relative growth rate (RGR) = P/A*T. Where A = mean dry weight of insect over unit time, E = dry weight of food consumed, F = dry weight of feces produced P = dry weight gain of insect, and T = duration of feeding period.

### Data analysis

Nutritional indices of *H. armigera* reared on different host plants were analyzed with one— way ANOVA using the statistical software Minitab ver. 14.0 (Minitab Inc. 1994) to find out similarities and significant differences. Statistical differences among the means were assessed using the LSD test at α = 0.05. Data were tested for normality before analysis.

A dendrogram of different host plants according to nutritional indices of whole larval instars (third, fourth, and fifth instars) of *H. armigera* on different host plants was created after cluster analysis by Ward's method using SPSS 16.0 statistical software.

## Results

The results of the nutritional indices of third, fourth, fifth, and whole larval instars of *H. armigera* are shown in Tables 1, 2, 3, and 4. Nutritional indices of the third instar larvae of *H. armigera* were significantly different on various host plants (*p* < 0.01). The larvae reared on potato Agria showed the highest value of ECD (*F* = 16.62; df = 10, 221; *p* < 0.01) (50.800 ± 0.104%). However, the lowest value of ECD was on chickpea Hashem (6.677 ± 0.007%). Also, the highest value of ECI (*F* = 5.34; df = 10, 231; *p* < 0.01) (13. 630 ± 0.016%) was on potato Agria compared with the other hosts. The larvae fed on white kidney bean Dehghan had the highest CI (*F* = 5.55; df = 10, 233; *p* < 0.01) and RCR (*F* = 17.76; df = 10, 230; *p* < 0.01) values (5.498 ± 0.353 and 1.833 ± 0.118, respectively). However, the lowest value of CI (2.753 ± 0.294) was observed on potato Agria (Table 1).

The highest (92.651 ± 0.004%) and lowest (57.140 ± 0.049%) AD values (*F* = 8.06; df = 10, 234; *p* < 0.01) of the fourth instar larvae of *H. armigera* were on chickpea Azad and potato Agria, respectively. The potato Satina and tomato Meshkin showed the highest and lowest values of RGR (*F* = 18. 6; df = 10, 244; *p* < 0.01) (0.328 ± 0.026 and 0.075 ± 0.006), respectively (Table 2).

The highest value of CI (3.717 ± 0.091) (*F* = 31. 22; df = 10, 242; *p* < 0.01) and the lowest values of ECI (4.832 ± 0.001%) (*F* = 16. 40; df = 10, 239; *p* < 0.01) and ECD (5.554 ± 0.002%) (*F* = 21. 50; df = 10, 238; *p* < 0.01) of fifth instar *H. armigera* were observed on tomato Meshkin (Table 3).

The results of Table 4 for whole larval instars (third, fourth, and fifth instars) showed that the ECI (*F* = 21.47; df = 10, 243; *p* < 0.01) and ECD (*F* = 23.16; df = 10, 226; *p* < 0.01) values were the highest on potatoes Satina (14.640 ± 0.014%) and Agria (21.380 ± 0.015%), respectively, and lowest on tomato Meshkin for both indices (ECI: 5.748 ± 0.002% and ECD: 7.341 ± 0.002%). The larvae fed on tomato Meshkin and potato Agria had the highest (3.955 ± 0.119) and lowest (1.983 ± 0.110) values of CI, respectively (*F* = 31.97; df = 10, 245; *p* < 0.01). The larvae fed on white kidney bean Dehghan and tomato Meshkin had the highest (1.528 ± 0.051) and lowest (0.988 ± 0.029) values of RCR, respectively (*F* = 18; df = 10, 245; *p* < 0.01). Our results indicated that the highest and lowest values of AD (*F* = 15. 48; df = 10, 240; *p* < 0.01) were on chickpea Azad (87.896 ± 0.007) and potato Agria (57.260 ± 0.037%), respectively.

Values of AD, ECI, and ECD among third, fourth, and fifth instars are compared in Table 5. In most cases, the highest and lowest values of AD (*F* = 7.48; df = 2, 116; *p* < 0.01) were on third and fifth instars, respectively. The highest and lowest values of ECI (*F* = 8.75; df = 2, 114; *p* < 0.01) and ECD (*F* = 15.56; df = 2, 113; *p* < 0.01) were on fourth and third instars, respectively.

**Figure 1.**
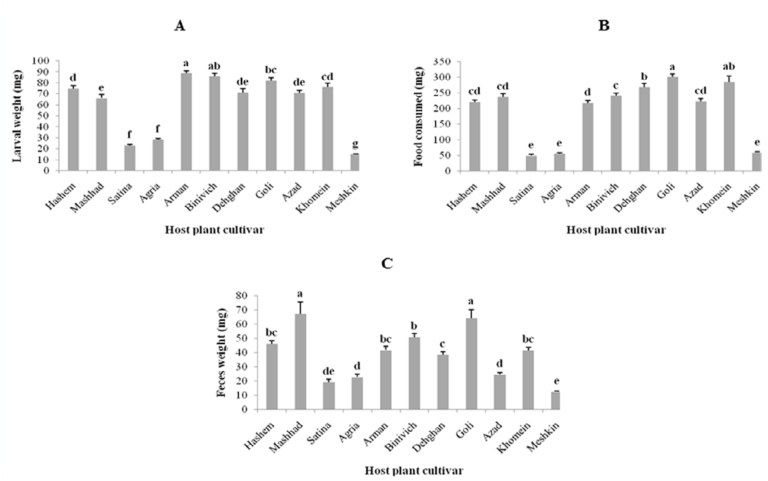
(A) Mean larval weight, (B) food consumed, and (C) feces produced of *Helicoverpa armigera* for whole larval instars on different host plants. Bars represent standard error of the means. The means followed by different letters are significantly different (LSD, *p* < 0.01 ). High quality figures are available online.

The larval weights, food consumed, and feces produced by the whole larval instars are shown in Figure 1. Larval weight (*F* = 157. 07; df = 10, 227; *p* < 0.01) and food consumed (*F* =153.95; df = 10, 244; *p* < 0.01) by the whole larval instars were highest on chickpea Arman (88.710 ± 2.120 mg) and red kidney bean Goli (301 ± 10.100 mg), respectively, and lowest on tomato Meshkin (15.205 ± 0.272 mg) and potato Satina (50.310 ± 3.970 mg), respectively. The highest and lowest values of feces produced (*F* = 27.97; df = 10, 239; *p* < 0.01) were on cowpea Mashhad (67.520 ± 8.160) and tomato Meshkin (12.663 ± 0.526), respectively.

**Figure 2.**
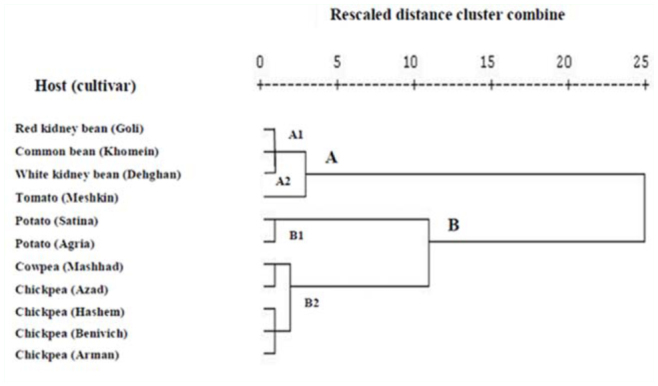
Dendrogram of different host plants according to nutritional indices of *Helicoverpa armigera* reared on different host plants (Ward's method). High quality figures are available online.

A dendrogram according to nutritional indices of whole larval instars of *H. armigera* reared on different host plants is shown in Figure 2. The dendrogram shows two clusters labeled ‘A’ (including subclusters A1 and A2) and ‘B’ (including subclusters B1 and B2). Different host plants were grouped within each cluster according to the comparison of the nutritional indices of *H. armigera* reared on the cultivars of various host plants. Cluster A included subclusters A1 (red kidney bean Goli, common bean Khomein, and white kidney bean Dehghan) as an intermediate group, and A2 (tomato Meshkin) as a partially unsuitable host; cluster B consisted of subclusters B1 (potatoes Satina and Agria) and B2 (cowpea Mashhad and chickpeas Azad, Hashem, Binivich, and Arman) as suitable hosts.

## Discussion

Applying resistant cultivars plays a key role in integrated pest management programs for any crop plant resistance to pests (Wilson and Huffaker 1976; Endo et al. 2007). The significant differences in the ability of insect larvae to utilize different host plants efficiently suggest some intrinsic variations among the plant species. The difference in survival and development of insects on different cultivars might have been caused by antibiotic effects, poor nutritional quality of the food, pericarp thickness, and/or secondary plant biochemicals (Singh et al. 1965; Sharma et al. 1982; Samraj and David 1988). It is generally accepted that low dietary protein can cause an increase in the rate at which larvae feed (Rausher 1981; Slansky 1993); conversely, a high protein diet can reduce feeding rates (Mattson 1980). According to Cohen and Patana (1984), although there was no difference in nitrogen content in the artificial and bean diets, *H. zea* larvae fed on the artificial diet had a much higher nitrogen content than the larvae reared on the bean diet, suggesting that larvae fed the artificial diet passed more material through their systems and accumulated more body nitrogen than did those feed beans.

The nutritional indices, particularly ECI and ECD values, of *H. armigera* reared on different host plants were significantly different, suggesting that the various host plants had different nutritional values. ECI is a general index of an insect's ability to use the food consumed for growth and development, and ECD is an index of the efficiency of conversion of digested food into growth (Nathan et al. 2005). The data of nutritional indices for the fourth and fifth instars of *H. armigera* are not consistent with each other. This is because the nutritional requirements of the insect change through development, and such differences typically result in changes in food consumption and feeding behavior (Barton Browne 1995). Typically, when the quantity of food ingested decreases, the duration of development is extended and the insect becomes smaller and lighter. Another reason may be related to increased instar duration when an increasing amount of ingested food must be allocated towards maintenance of metabolism. It is likely due to the fact that nutritional requirements would be positively correlated with the mass of the insect (Phillipson 1981; Schroeder 1981). The leaves were used for feeding first and second instars larvae, and the pods and fruit for feeding the third to fifth instars. Since older larvae of Lepidoptera have a greater protein need (Simpson et al. 1988), the nutritional requirements of penultimate and ultimate instars were different.

An important fitness indicator of insect population dynamics is body weight (Liu et al. 2004). The larval weight in the whole larval instars was the highest on chickpea Arman and lowest on tomato Meshkin. Naseri et al. (2010) showed that the larval weight of *H. armigera* is affected by different soybean varieties. The present finding on the larval weight of *H. armigera* reared on tomato Meshkin (15.205 ± 0.272 mg) was similar to that reported by Naseri et al. (2010) on L17 (15.497 ± 0.911 mg).

Among different host plants, the highest CI value of the whole larval instars of *H. armigera* was on tomato Meshkin (3.955 ± 0.119), indicating that the rate of intake relative to the mean larval weight during the feeding period was the highest on this host. This value was similar to that reported by Naseri et al. (2010) on variety 356 (4.022 ± 0.870). The results for AD value of the whole larval instars of *H. armigera* fed on cowpea Mashhad (69.530 ± 0.043%), tomato Meshkin (67.470 ± 0.016%), and potato Satina (66.640 ± 0.023%) were nearly similar to those reported by Naseri et al. (2010) on Clark (69.900 ± 0.047%) and Sahar (65.900 ± 0.061%), and Ashfaq et al. (2003) on *Sorghum vulgaris* (69.33%) and *Gossypium* *hirsutum* var. NIAB-98 (66.15%). Wang et al. (2006) indicated that AD value of *H. armigera* was 21.372 ± 0.013% on an artificial diet.

For the whole larval instars, the highest ECI and ECD values were on the potatoes Satina and Agria, suggesting that the larvae were more efficient at conversion of ingested and digested food to body biomass with a high increase in larval weight. Despite the tomato Meshkin having the highest CI value, it also had the lowest values of ECI and ECD (Table 4), indicating that larvae feeding on this host were less effective in converting ingested and digested food to biomass. It is well known that the degree of food utilization depends on the digestibility of food, and the efficiency with which digested food is converted into biomass (Batista Pereira et al. 2002). The mean ECD value of whole larval instars reared on different host plants (13.138 ± 0.008%) was lower than that reported by Wang et al. (2006) on an artificial diet (41.200 ± 0.012%). Such differences in ECD values between our work and previous studies is due to the use of artificial diets in those studies, which are designed to provide complete nutrition for high insect performance, and are considered to be better than natural diets (Hari et al. 2007).

Overall, lepidopteran larvae fed on high— nutrient food increase growth rates and complete the development period faster than larvae fed on low—nutrient food (Hwang et al. 2008). The duration of the feeding period is an effective factor in the RGR and RCR values. Our results of whole larval instars showed that the RCR and RGR values were the highest on white kidney bean Dehghan and potato Satina, respectively, and lowest on tomato Meshkin. Our results indicated that the tomato Meshkin was a low—nutrient food for the larvae, and a longer period of development was therefore necessary to complete immature stages. Conversely, the white kidney bean Dehghan and potato Satina were high—nutrient foods for the larvae, and a shorter period of development was needed to complete immature stages.

For the whole larval instars, tomato Meshkin showed the lowest ECD and ECI values, possibly due to the lack of nutritional components and the presence of some secondary chemicals. Kotkar et al. (2009) reported that legumes such as chickpea, pigeon pea, and pea had the highest protein content, and tomato had very low protein content. Also, tomato itself is not a fine host plant for *H. armigera* larvae, as previous works have shown (Liu et al. 2004). Banerjee and Kalloo (1989) reported a significant negative correlation between ortho—dihydroxy phenols in tomato leaves and larval feeding rate. Tomato acidity may be negatively correlated with larval feeding (Kashyap and Verma 1987). This supports the suggestion that the tomato Meshkin is a less suitable host plant for *H. armigera* larvae than the others.

Third instar larvae fed on chickpea Hashem had the highest AD and almost the lowest ECD, in agreement with Martin and Pulin (2004), who reported that larvae *Lycaena dispar* fed on *Rumex obtusifolius* had highest AD and lowest ECD compared with other hosts. Apparently, the increase in AD could not compensate for the decrease in ECD, which accordingly led to a reduced growth rate. Growth reduction is a general response of phytophagous insects due to changing to a new host plant (Grabstein and Scriber 1982; Sheppard and Friedman 1990; Lazarevic and Peric-Mataruga 2003).

The relationship between *H. armigera* digestive enzymes and the nutrient composition of the different diets reveals the adaptive nature of the polyphagous pest. *Helicoverpa armigera* gut amylase and proteinase levels are balanced based on diet composition and larval developmental stages (Kotkar et al. 2009). The results presented in Table 5 for comparison of the ECI and ECD values showed that these values, in most cases, increased from third to fourth instar and then decreased from fourth to fifth (ultimate) instar. The general trend of increases in ECD from early to late instars was reported by Slansky and Scriber (1985). Physiological changes among penultimate and ultimate instar larvae reared on different host plants are perhaps partially responsible for the differences in such decreases in ECD and ECI values (Nation 2001). Physiological changes in the nervous system of the fifth instar causes termination of feeding, induced wandering behavior, and metabolic changes that occur in the fat body. Because of such physiological and behavioral changes, the nutritional responses (particularly ECD and ECI) of these two larval instars were different. Also, the other major reason for these differences could be a result of changes at the levels of digestive enzymes. Patankar et al. (2001) showed that *H. armigera* midgut proteinase levels reached a maximum in the penultimate instar and were decreased in the ultimate instar. In the case of *H. armigera*, maximum food intake occurs during the penultimate instar, and feeding slows down or stops in the ultimate instar. The highest ECI and ECD values in the fourth instar indicated a higher efficiency of the conversion of ingested and digested food to body biomass. Hence, this instar could potentially cause damage on the host plants; control of *H. armigera* should be considered before fourth instar.

The results of the cluster analysis represented here indicated that grouping within each cluster might be due to a high level of physiological similarity of different host plants. The results of the comparative nutritional indices of *H. armigera* on different host plants revealed that subcluster A2 was the least suitable and subcluster B1 was the most suitable for *H. armigera.* However, the hosts in subcluster A1 had an intermediate status. These results were associated with ECI and ECD values of whole larval instars on different host plants. According to Table 4, the ECI and ECD values of the whole larval instars were the highest on potatoes Satina and Agria and lowest on tomato Meshkin compared to the others. Our observations on the life history and fecundity of *H. armigera* reared on different host plants indicated that the longest development time, the highest percentage mortality of immature stages, lowest daily fecundity (eggs per reproduction day), and total fecundity (eggs during reproduction period) were on tomato Meshkin (data not shown), which are in agreement with the current research regarding nutritional indices (especially ECI and ECD) of whole larval instars on this host. The results related to unsuitability of the tomato Meshkin as a host is in agreement with the findings of Liu et al. (2004), who reported that the suitability of host plants is classified as follows (descending in suitability): cotton, corn, legume, tobacco, tomato, and hot pepper.

Analysis of nutritional indices can lead to understanding of the behavioral and physiological basis of insect response to host plants (Lazarevic and Peric-Mataruga 2003). Lower fitness of *H. armigera* on some host plants may be due to the presence of some secondary phytochemicals in these host plants, or absence of primary nutrients necessary for growth and development. To obtain more applicable information for *H. armigera* control, more attention should be devoted to study demographic parameters of this pest under laboratory and field conditions, as well as to investigate its nutritional indices on different host plants under field conditions.

**Table 1.**
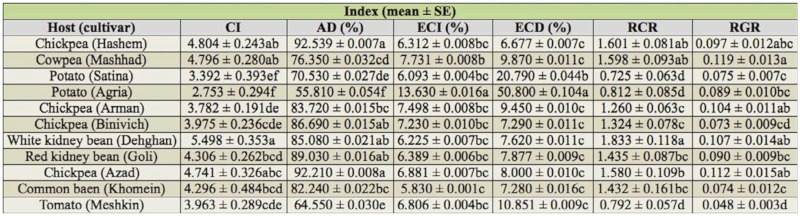
Nutritional indices of third instar larvae of *Helicoverpa armigera* on different host plants.

**Table 2.**
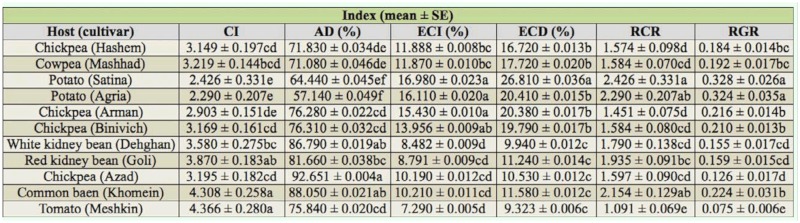
Nutritional indices of fourth instar larvae of *Helicoverpa armigera* on different host plants.

**Table 3.**
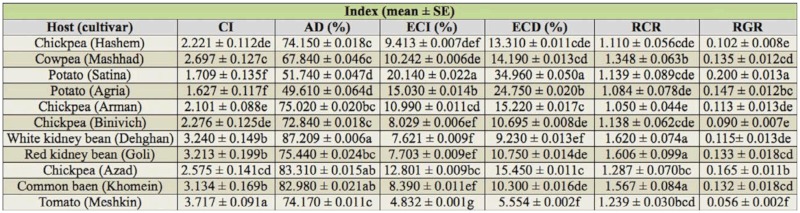
Nutritional indices of fifth instar larvae of *Helicoverpa armigera* on different host plants.

**Table 4.**
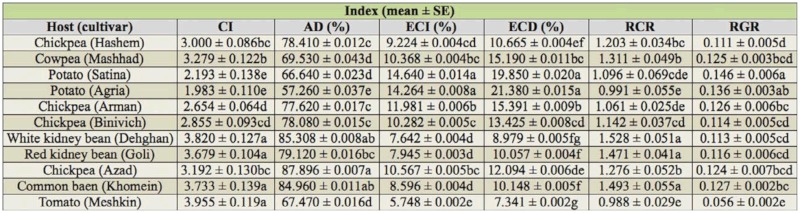
Nutritional indices of whole larval instars of * Helicoverpa armigera* on different host plants.

**Table 5.**
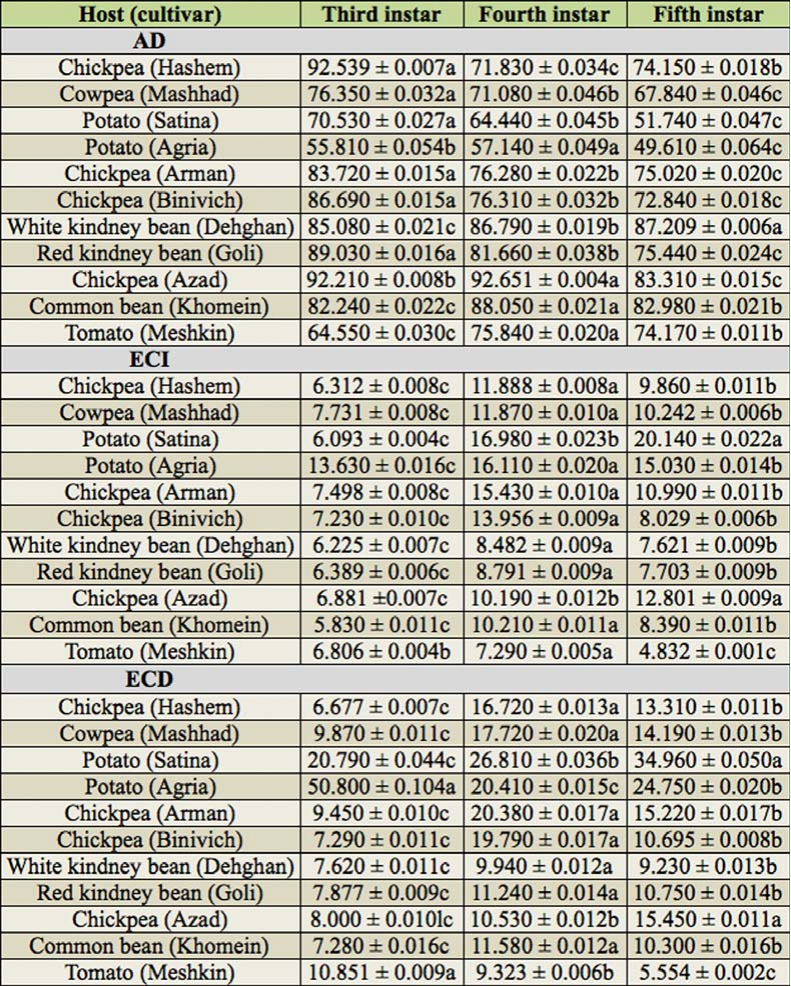
Comparison of approximate digestibility (AD), efficiency of conversion of ingested food (ECI), and efficiency of conversion of digested food (ECD) among third, fourth, and fifth instars of *Helicoverpa armigera* reared on different host plants.
